# Individualized therapy development for rare diseases: individualized at every step of the way

**DOI:** 10.1177/26330040251386869

**Published:** 2025-10-18

**Authors:** Marlen C. Lauffer, Tim Yu, Annemieke Aarstma-Rus

**Affiliations:** Dutch Center for RNA Therapeutics, Department of Human Genetics, Leiden University Medical Center, The Netherlands; N=1 Collaborative, 7 Carole Place, Somerville, MA 02143, USA; Boston Children’s Hospital, Boston, MA, USA; N=1 Collaborative, 7 Carole Place, Somerville, MA 02143, USA; Dutch Center for RNA Therapeutics, Department of Human Genetics, Leiden University Medical Center, Albinusdreef 2, 2333 ZA Leiden, The Netherlands, N=1 Collaborative, 7 Carole Place, Somerville, MA 02143, USA

**Keywords:** genetic therapies, individualized therapy, precision medicine

In 2018, the first-ever individualized genetic therapy, developed for a girl with *CLN7* Batten’s disease, was reported.^1^ Individualized genetic therapies precisely target the cause of disorders at the level of the gene, transcript, or variant.^2^ The therapy used for this Batten’s disease patient was an antisense oligonucleotide (ASO) able to block mis-splicing caused by one of the patient’s *CLN7* *(MFSD8)* pathogenic variants.

To date, over 20 individualized ASOs have been developed and delivered to over 30 patients worldwide, with new cases being added regularly.^3^ Notably, the number of individualized oligonucleotide therapies has now surpassed the number of commercially available oligonucleotide therapies, highlighting the substantial efforts made in this space in recent years. There have also been reports of individualized gene addition and base editing therapies,^4,5^ underlining that the field of individualized treatment is expanding rapidly.

Importantly, models for developing individualized therapies substantially differ from those for commercial drugs. The IRDiRC N-of-1 task force has released a roadmap detailing the steps, processes, and infrastructure aspects involved.^6^ While each specific therapy may be individualized, there are common steps and processes that apply to all.

Nonprofit initiatives like the N=1 Collaborative (N1C) aim to streamline development processes and provide standardized protocols, frameworks, and guidelines to help those developing individualized therapies.^3^ N1C also gathers information and expertise gained from each therapeutic journey, both successful and unsuccessful, to improve and advise the development of future individualized treatments. While guidelines and roadmaps are being built, it is clear they will be fine-tuned and updated when the field develops further.

For this special edition, we asked authors to share their experiences with individualized therapy development across the development journey. The articles reflect the challenges encountered across the different steps.

The first step in the therapeutic journey is obtaining a genetic diagnosis, which is often lacking for patients with ultra-rare genetic diseases. Battaglia et al.^7^ report a case of a female presenting with intellectual disability and microcephaly during early development who developed pilomatricomas, a type of rare benign skin tumor, at the age of 17 years. This form of skin tumor is associated with very few distinct genetic syndromes and, following earlier negative molecular diagnosis, prompted the team to perform whole exome sequencing, resulting in the identification of a pathogenic variant in the *EP300* gene leading to a diagnosis of Rubinstein–Taybi syndrome 2.

How treatment needs to be personalized and adapted to the patient’s anatomy was reported by Frijns et al.^8^ who describe two congenitally deaf siblings diagnosed with likely pathogenic variants in the *FGF3* gene associated with labyrinthine aplasia, microtia, and microdontia (LAMM) syndrome. The team opted for an auditory brainstem implant in the two boys to target the deafness and noted that the anatomy of the brainstem in LAMM syndrome is abnormal, which led them to adjust standard procedures leading to a better outcome for the patients. The authors stress that, given anatomical differences across different genetic syndromes, one should adjust surgeries and other procedures to account for said differences.

To measure treatment efficacy, clinical outcome measures need to be established for each case. Müller et al.^9^ provide a review of outcome measures used in clinical trials for neurodevelopmental disorders and intellectual disability. The team demonstrates that currently, a plethora of outcome measures are being used without standardization, prompting the authors to stress the importance of building consensus on common measures. The authors further highlight to measure what is relevant to patients to ensure clinical benefit for the target individual and group. Especially for individualized therapeutic developments, the patient matters most and should be involved in developing clinical outcome measures.

Lastly, authors from the Dutch Center for RNA Therapeutics,^10^ a nonprofit consortium in the Netherlands aiming to develop individualized RNA therapies for individuals diagnosed with rare disorders of the brain and eye, describe the establishment of their center, the hurdles and challenges the team had to face and still encounters, and the lessons they have learned so far to support others in setting up similar initiatives.

The challenge that remains is moving from n-of-1 for a few cases to n-of-1 for many cases. Here, we need to work together in interdisciplinary teams with regulators, ethicists, clinicians, researchers, and the patients to ensure these efforts can be scaled and are sustainable.^6^

## Declarations

**Ethics approval and consent to participate:** Not applicable.

**Consent for publication:** Not applicable.

**Availability of data and materials:** Not applicable.
